# Risk of Glioblastoma Multiforme in Patients Taking Ion Channel Blockers

**DOI:** 10.7759/cureus.30277

**Published:** 2022-10-13

**Authors:** David R Hallan, Cyril S Tankam, Thaddeus Harbaugh, Elias Rizk

**Affiliations:** 1 Neurosurgery, Penn State Health Milton S. Hershey Medical Center, Hershey, USA

**Keywords:** ion channel blockers, glioblastoma multiforme, gbm, database, ion channel, neurosurgery, glioblastoma

## Abstract

Background

Ion channels play a role in the development and progression of glioblastoma multiforme. This study investigates the association between the risk of developing glioblastoma multiforme in patients taking these medications.

Methods

A retrospective propensity score-matched analysis was performed using the TriNetX multinational electronic health record database for patients taking verapamil, digoxin, amiodarone, or diltiazem versus those not taking these medications. The outcome of interest was the incidence of glioblastoma multiforme.

Results

Verapamil users had an OR of 0.494 (p < 0.0001) of developing glioblastoma versus verapamil non-users. Patients on digoxin had an OR of 0.793 (p = 0.2393), patients on amiodarone had an OR of 0.600 (p = 0.0035), patients on diltiazem had an OR of 0.584 (p < 0.0001), and patients on verapamil, digoxin, amiodarone, or diltiazem had an OR of 0.641 (p < 0.0001) of developing glioblastoma versus patients not taking these medications.

Conclusion

In patients taking the ion channel blockers diltiazem, amiodarone, or verapamil, the odds of developing glioblastoma multiforme were lower than in patients not taking these medications.

## Introduction

Glioblastoma multiforme (GBM) is a primary central nervous system (CNS) tumor that accounts for approximately 16% of all CNS neoplasms and causes approximately 15,000 deaths yearly in the United States [1,2]. The current school of thought suggests that this malignancy originates from the supporting cells of the CNS, known as glial cells. This tumor exhibits a particularly high affinity for invasion and spread into the surrounding brain parenchyma. Although current surgical and medical therapy is available for managing this tumor, the prognosis for patients remains dismal [3]. Only two factors have been shown to increase the risk of developing gliomas: high doses of ionizing radiation and certain inherited mutations [4]. However, several factors have been shown to worsen the prognosis of GBM, one of them being mutations in ion channels, specifically sodium, potassium, and calcium transporters, as well as the sodium/potassium-ATPase. Recent studies have shown that gliomas use these ion channels to foster their growth and invasion of the brain [5]. As such, it is possible to hypothesize that ion channel blockers could play a role in the development and progression of GBM.

This study investigates the association between the risk of developing GBM in patients taking ion channel blockers, specifically verapamil, diltiazem, digoxin, and amiodarone.

## Materials and methods

This study is a retrospective case-control study model using a multi-institutional healthcare database, the TriNetX research network, to collect data on patients diagnosed with GBM while taking verapamil, digoxin, diltiazem, or amiodarone. The TriNetX research network is a database that houses de-identified electronic medical records from several healthcare organizations spanning 57 academic medical institutions, and the information in this database is updated daily. This database contains information regarding patient demographics, diagnoses, medications, and outcomes. Since the database is federated, an Institutional Review Board approval for this study has been waived.

The TriNetX database was interrogated for patients who took the ion channel blockers verapamil, digoxin, amiodarone, or diltiazem. These were stratified into four different groups. The primary outcome of interest was the risk of GBM development in patients taking one of these drugs compared to patients who were not taking the drug. An analysis was performed for each of the drugs individually without patients taking any other ion channel blockers, as well as in a combined cohort where patients could be on any ion channel blocker. Chi-square analysis was used for categorical variables. The significance level was set as p-value ≤ 0.05.

## Results

Tables 1, 2 show the baseline characteristics and measures of association for our patients taking verapamil. After matching, of the 512,098 patients using verapamil, 45 patients (0.009%) subsequently developed a glioblastoma. This is in comparison to the 512,098 patients not taking verapamil, of which 91 patients (0.018%) developed a glioblastoma (p = <0.0001, odds ratio (OR) = 0.494, 95% confidence interval (CI) = 0.346, 0.707).

**Table 1:** Baseline characteristics for patients taking verapamil

Cohort 1 (N = 522,713) and cohort 2 (N = 6,437,822) characteristics before propensity score matching											
	Demographics										
		Cohort		Code		Mean ± SD	Patients	% of cohort		P-value	Std. diff.
		1		AI	Age at index	60.1 ± 15.8	512,098	100%		<0.001	0.822
		2				44.0 ± 22.8	6,437,658	100%			
		1		2106-3	White		360,872	70.50%		<0.001	0.067
		2					4,335,933	67.40%			
		1		2054-5	Black or African American		77,336	15.10%		<0.001	0.079
		2					1,161,646	18.00%			
		1		M	Male		243,685	47.60%		<0.001	0.126
		2					2,662,613	41.40%			
	Diagnosis										
		Cohort		Code		Mean ± SD	Patients	% of cohort		P-value	Std. diff.
		1		K74	Fibrosis and cirrhosis of the liver		8,621	1.70%		<0.001	0.066
		2					60,006	0.90%			
		1		I10-I16	Hypertensive diseases		248,178	48.50%		<0.001	0.476
		2					1,677,156	26.10%			
		1		E08-E13	Diabetes mellitus		105,204	20.50%		<0.001	0.229
		2					780,400	12.10%			
		1		N17-N19	Acute kidney failure and chronic kidney disease		71,413	13.90%		<0.001	0.281
		2					364,519	5.70%			
		1		F17	Nicotine dependence		62,388	12.20%		<0.001	0.154
		2					488,350	7.60%			
		1		F10.1	Alcohol abuse		11,819	2.30%		<0.001	0.067
		2					90,056	1.40%			
		1		J40-J47	Chronic lower respiratory diseases		94,392	18.40%		<0.001	0.132
		2					876,601	13.60%			
		1		I48	Atrial fibrillation and flutter		57,620	11.30%		<0.001	0.304
		2					220,156	3.40%			
		1		I50	Heart failure		71,447	14.00%		<0.001	0.371
		2					233,409	3.60%			
		1		I20-I25	Ischemic heart diseases		157,034	30.70%		<0.001	0.642
		2					438,602	6.80%			
Cohort 1 (N = 512,098) and cohort 2 (N = 512,098) characteristics after propensity score matching											
	Demographics										
		Cohort		Code		Mean ± SD	Patients	% of cohort		P-value	Std. diff.
		1		AI	Age at index	60.1 ± 15.8	512,098	100%		<0.001	0.013
		2				60.3 ± 15.8	512,098	100%			
		1		2106-3	White		360,872	70.50%		0.061	0.004
		2					361,736	70.60%			
		1		2054-5	Black or African American		77,336	15.10%		0.755	0.001
		2					77,449	15.10%			
		1		M	Male		243,685	47.60%		0.49	0.001
		2					243,336	47.50%			
	Diagnosis										
		Cohort		Code		Mean ± SD	Patients	% of cohort		P-value	Std. diff.
		1		K74	Fibrosis and cirrhosis of the liver		8,621	1.70%		0.812	<0.001
		2					8,590	1.70%			
		1		I10-I16	Hypertensive diseases		248,178	48.50%		0.28	0.002
		2					248,724	48.60%			
		1		E08-E13	Diabetes mellitus		105,204	20.50%		0.365	0.002
		2					105,575	20.60%			
		1		N17-N19	Acute kidney failure and chronic kidney disease		71,413	13.90%		0.677	0.001
		2					71,267	13.90%			
		1		F17	Nicotine dependence		62,388	12.20%		0.022	0.005
		2					61,633	12.00%			
		1		F10.1	Alcohol abuse		11,819	2.30%		<0.001	0.011
		2					11,023	2.20%			
		1		J40-J47	Chronic lower respiratory diseases		94,392	18.40%		0.986	<0.001
		2					94,399	18.40%			
		1		I48	Atrial fibrillation and flutter		57,620	11.30%		0.026	0.004
		2					56,912	11.10%			
		1		I50	Heart failure		71,447	14.00%		<0.001	0.011
		2					69,468	13.60%			
		1		I20-I25	Ischemic heart diseases		157,034	30.70%		0.001	0.006
		2					155,516	30.40%			

**Table 2:** Measures of association for patients taking verapamil and glioblastoma development

Cohort				Patients in cohort	Patients with outcome		Risk
1		Verapamil		512,098	45		0.01%
2		No verapamil		512,098	91		0.02%
				95% CI		z	p
Risk difference			-0.01%	(-0.013, -0.005)		-3.945	<0.0001
Risk ratio			0.495	(0.346, 0.707)			
Odds ratio			0.494	(0.346, 0.707)			

Tables 3, 4 show the baseline characteristics and measures of association for our patients taking digoxin. Of the 400,800 patients using digoxin, 46 patients (0.011%) developed a glioblastoma, while of the 400,800 patients not taking digoxin, 58 patients (0.014%) did (p = 0.2393, OR = 0.793, 95% CI = 0.539, 1.168).

**Table 3:** Baseline characteristics for patients taking digoxin

Cohort 1 (N = 441,710) and cohort 2 (N = 6,488,498) characteristics before propensity score matching												
	Demographics											
		Cohort		Code		Mean ± SD		Patients	% of cohort		P-value	Std. diff.
		1		AI	Age at index	67.8 ± 17.3		424,025	100%		<0.001	1.178
		2				44.0 ± 22.7		6,488,324	100%			
		1		2106-3	White			301,763	71.20%		<0.001	0.083
		2						4,368,586	67.30%			
		1		2054-5	Black or African American			43,147	10.20%		<0.001	0.23
		2						1,175,846	18.10%			
		1		M	Male			228,788	54.00%		<0.001	0.255
		2						2,679,748	41.30%			
	Diagnosis											
		Cohort		Code		Mean ± SD		Patients	% of cohort		P-value	Std. diff.
		1		K74	Fibrosis and cirrhosis of the liver			6,821	1.60%		<0.001	0.059
		2						61,241	0.90%			
		1		I10-I16	Hypertensive diseases			178,905	42.20%		<0.001	0.337
		2						1,714,988	26.40%			
		1		E08-E13	Diabetes mellitus			129,006	30.40%		<0.001	0.23
		2						1,328,705	20.50%			
		1		N17-N19	Acute kidney failure and chronic kidney disease			81,736	19.30%		<0.001	0.192
		2						797,676	12.30%			
		1		F17	Nicotine dependence			78,807	18.60%		<0.001	0.404
		2						367,245	5.70%			
		1		F10.1	Alcohol abuse			33,130	7.80%		0.026	0.004
		2						500,814	7.70%			
		1		J40-J47	Chronic lower respiratory diseases			8,977	2.10%		<0.001	0.054
		2						91,540	1.40%			
		1		I48	Atrial fibrillation and flutter			78,837	18.60%		<0.001	0.133
		2						889,710	13.70%			
		1		I50	Heart failure			169,820	40.00%		<0.001	1.008
		2						197,088	3.00%			
		1		I20-I25	Ischemic heart diseases			123,790	29.20%		<0.001	0.744
		2						222,074	3.40%			
Cohort 1 (N = 400,800) and cohort 2 (N = 400,800) characteristics after propensity score matching												
	Demographics											
		Cohort		Code		Mean ± SD		Patients	% of cohort		P-value	Std. diff.
		1		AI	Age at index	67.1 ± 17.4		400,800	100%		<0.001	0.053
		2				68.0 ± 16.8		400,800	100%			
		1		2106-3	White			285,025	71.10%		<0.001	0.008
		2						286,563	71.50%			
		1		2054-5	Black or African American			41,969	10.50%		<0.001	0.012
		2						43,428	10.80%			
		1		M	Male			215,191	53.70%		<0.001	0.017
		2						218,660	54.60%			
	Diagnosis											
		Cohort		Code		Mean ± SD		Patients	% of cohort		P-value	Std. diff.
		1		K74	Fibrosis and cirrhosis of the liver			6,405	1.60%		0.245	0.003
		2						6,275	1.60%			
		1		I10-I16	Hypertensive diseases			165,032	41.20%		<0.001	0.03
		2						171,031	42.70%			
		1		E08-E13	Diabetes mellitus			120,504	30.10%		<0.001	0.033
		2						126,550	31.60%			
		1		N17-N19	Acute kidney failure and chronic kidney disease			76,156	19.00%		<0.001	0.03
		2						81,008	20.20%			
		1		F17	Nicotine dependence			70,372	17.60%		0.005	0.006
		2						71,325	17.80%			
		1		F10.1	Alcohol abuse			31,294	7.80%		0.003	0.007
		2						30,582	7.60%			
		1		J40-J47	Chronic lower respiratory diseases			8,328	2.10%		<0.001	0.01
		2						7,739	1.90%			
		1		I48	Atrial fibrillation and flutter			71,933	17.90%		0.984	<0.001
		2						71,926	17.90%			
		1		I50	Heart failure			146,884	36.60%		<0.001	0.015
		2						144,007	35.90%			
		1		I20-I25	Ischemic heart diseases			104,670	26.10%		<0.001	0.015
		2						102,127	25.50%			

**Table 4:** Measures of association for patients taking digoxin and glioblastoma development

Cohort				Patients in cohort	Patients with outcome	Risk
1		Digoxin		400,800	46	0.01%
2		No digoxin		400,800	58	0.01%
				95% CI	z	p
Risk difference			-0.003%	(-0.008, 0.002)	-1.177	0.239
Risk ratio			0.793	(0.539, 1.168)		
Odds ratio			0.793	(0.539, 1.168)		

Tables 5, 6 show the baseline characteristics and measures of association for our patients taking amiodarone. A total of 543,288 patients were taking amiodarone; of this number, 51 patients (0.009%) developed a glioblastoma. A matched group of 543,288 patients not taking amiodarone was identified and, in this cohort, 85 patients (0.016%) developed a glioblastoma (p = 0.0035, OR = 0.6, 95% CI = 0.424, 0.849).

**Table 5:** Baseline characteristics for patients taking amiodarone

Cohort 1 (N = 646,598) and cohort 2 (N = 6,413,181) characteristics before propensity score matching												
	Demographics											
		Cohort		Code		Mean ± SD		Patients	% of cohort		P-value	Std. diff.
		1		AI	Age at index	68.2 ± 13.9		642,771	100%		<0.001	1.301
		2				43.7 ± 22.7		6,413,010	100%			
		1		2106-3	White			458,481	71.30%		<0.001	0.089
		2						4,311,815	67.20%			
		1		2054-5	Black or African American			71,752	11.20%		<0.001	0.199
		2						1,164,000	18.20%			
		1		M	Male			398,131	61.90%		<0.001	0.429
		2						2,628,514	41.00%			
	Diagnosis											
		Cohort		Code		Mean ± SD		Patients	% of cohort		P-value	Std. diff.
		1		K74	Fibrosis and cirrhosis of the liver			12,352	1.90%		<0.001	0.084
		2						59,755	0.90%			
		1		I10-I16	Hypertensive diseases			329,918	51.30%		<0.001	0.537
		2						1,670,564	26.00%			
		1		E08-E13	Diabetes mellitus			152,108	23.70%		<0.001	0.306
		2						774,882	12.10%			
		1		N17-N19	Acute kidney failure and chronic kidney disease			166,302	25.90%		<0.001	0.587
		2						346,303	5.40%			
		1		F17	Nicotine dependence			64,200	10.00%		<0.001	0.082
		2						491,406	7.70%			
		1		F10.1	Alcohol abuse			15,383	2.40%		<0.001	0.073
		2						89,828	1.40%			
		1		J40-J47	Chronic lower respiratory diseases			124,451	19.40%		<0.001	0.154
		2						876,350	13.70%			
		1		I48	Atrial fibrillation and flutter			271,736	42.30%		<0.001	1.073
		2						177,783	2.80%			
		1		I50	Heart failure			190,200	29.60%		<0.001	0.762
		2						206,561	3.20%			
		1		I20-I25	Ischemic heart diseases			250,057	38.90%		<0.001	0.835
		2						423,919	6.60%			
Cohort 1 (N = 543,288) and cohort 2 (N = 543,288) characteristics after propensity score matching												
	Demographics											
		Cohort		Code		Mean ± SD		Patients	% of cohort		P-value	Std. diff.
		1		AI	Age at index	67.1 ± 14.2		543,288	100%		<0.001	0.11
		2				68.6 ± 14.1		543,288	100%			
		1		2106-3	White			384,058	70.70%		<0.001	0.008
		2						386,072	71.10%			
		1		2054-5	Black or African American			62,661	11.50%		0.219	0.002
		2						63,071	11.60%			
		1		M	Male			329,165	60.60%		0.001	0.006
		2						330,885	60.90%			
	Diagnosis											
		Cohort		Code		Mean ± SD		Patients	% of cohort		P-value	Std. diff.
		1		K74	Fibrosis and cirrhosis of the liver			9,713	1.80%		0.06	0.004
		2						9,974	1.80%			
		1		I10-I16	Hypertensive diseases			255,101	47.00%		<0.001	0.032
		2						263,759	48.50%			
		1		E08-E13	Diabetes mellitus			118,383	21.80%		<0.001	0.026
		2						124,236	22.90%			
		1		N17-N19	Acute kidney failure and chronic kidney disease			116,493	21.40%		<0.001	0.016
		2						112,883	20.80%			
		1		F17	Nicotine dependence			51,538	9.50%		<0.001	0.009
		2						50,106	9.20%			
		1		F10.1	Alcohol abuse			12,059	2.20%		0.603	0.001
		2						12,139	2.20%			
		1		J40-J47	Chronic lower respiratory diseases			95,383	17.60%		<0.001	0.013
		2						98,004	18.00%			
		1		I48	Atrial fibrillation and flutter			176,728	32.50%		<0.001	0.063
		2						160,853	29.60%			
		1		I50	Heart failure			125,441	23.10%		<0.001	0.014
		2						122,234	22.50%			
		1		I20-I25	Ischemic heart diseases			177,864	32.70%		<0.001	0.014
		2						181,496	33.40%			

**Table 6:** Measures of association for patients taking amiodarone and glioblastoma development

Cohort		Patients in cohort	Patients with outcome	Risk
1	Amiodarone	543,288	51	0.01%
2	No amiodarone	543,288	85	0.02%
		95% CI	z	p
Risk difference	-0.01%	(-0.01, -0.002)	-2.916	0.0035
Risk ratio	0.6	(0.424, 0.849)		
Odds ratio	0.6	(0.424, 0.849)		

Tables 7, 8 show the baseline characteristics and measures of association for our patients taking diltiazem. A total of 828,618 patients were identified to have been taking diltiazem, and of this number, 94 patients (0.011%) developed a glioblastoma. In a matched group of 828,618 patients not taking diltiazem, 161 patients (0.019%) developed a glioblastoma (p < 0.0001, OR = 0.584, 95% CI = 0.453, 0.753).

**Table 7:** Baseline characteristics for patients taking diltiazem

Cohort 1 (N = 895,591) and cohort 2 (N = 6,361,272) characteristics before propensity score matching												
	Demographics											
		Cohort		Code		Mean ± SD		Patients	% of cohort		P-value	Std. diff.
		1		AI	Age at index	66.4 ± 14.7		879,921	100%		<0.001	1.19
		2				43.6 ± 22.7		6,361,111	100%			
		1		2106-3	White			640,364	72.80%		<0.001	0.121
		2						4,276,040	67.20%			
		1		2054-5	Black or African American			110,409	12.50%		<0.001	0.155
		2						1,151,481	18.10%			
		1		M	Male			405,529	46.10%		<0.001	0.095
		2						2,630,906	41.40%			
	Diagnosis											
		Cohort		Code		Mean ± SD		Patients	% of cohort		P-value	Std. diff.
		1		K74	Fibrosis and cirrhosis of the liver			10,925	1.20%		<0.001	0.03
		2						59,209	0.90%			
		1		I10-I16	Hypertensive diseases			388,689	44.20%		<0.001	0.395
		2						1,633,614	25.70%			
		1		E08-E13	Diabetes mellitus			156,196	17.80%		<0.001	0.162
		2						762,610	12.00%			
		1		N17-N19	Acute kidney failure and chronic kidney disease			128,695	14.60%		<0.001	0.31
		2						345,520	5.40%			
		1		F17	Nicotine dependence			73,433	8.30%		<0.001	0.027
		2						484,719	7.60%			
		1		F10.1	Alcohol abuse			17,819	2.00%		<0.001	0.05
		2						87,952	1.40%			
		1		I48	Atrial fibrillation and flutter			165,911	18.90%		<0.001	0.148
		2						854,137	13.40%			
		1		I50	Heart failure			230,044	26.10%		<0.001	0.708
		2						171,025	2.70%			
		1		I20-I25	Ischemic heart diseases			112,697	12.80%		<0.001	0.35
		2						216,558	3.40%			
Cohort 1 (N = 828,618) and cohort 2 (N = 828,618) characteristics after propensity score matching												
	Demographics											
		Cohort		Code		Mean ± SD		Patients	% of cohort		P-value	Std. diff.
		1		AI	Age at index	65.6 ± 14.7		828,618	100%		<0.001	0.044
		2				66.3 ± 14.8		828,618	100%			
		1		2106-3	White			599,855	72.40%		<0.001	0.012
		2						595,428	71.90%			
		1		2054-5	Black or African American			107,250	12.90%		<0.001	0.016
		2						111,823	13.50%			
		1		M	Male			381,608	46.10%		<0.001	0.03
		2						394,062	47.60%			
	Diagnosis											
		Cohort		Code		Mean ± SD		Patients	% of cohort		P-value	Std. diff.
		1		K74	Fibrosis and cirrhosis of the liver			10,269	1.20%		0.338	0.001
		2						10,133	1.20%			
		1		I10-I16	Hypertensive diseases			350,863	42.30%		<0.001	0.017
		2						357,943	43.20%			
		1		E08-E13	Diabetes mellitus			143,869	17.40%		<0.001	0.02
		2						150,221	18.10%			
		1		N17-N19	Acute kidney failure and chronic kidney disease			112,067	13.50%		<0.001	0.016
		2						116,736	14.10%			
		1		F17	Nicotine dependence			68,443	8.30%		<0.001	0.014
		2						65,296	7.90%			
		1		F10.1	Alcohol abuse			16,283	2.00%		<0.001	0.009
		2						15,310	1.80%			
		1		I48	Atrial fibrillation and flutter			148,156	17.90%		<0.001	0.007
		2						145,875	17.60%			
		1		I50	Heart failure			179,559	21.70%		<0.001	0.044
		2						164,661	19.90%			
		1		I20-I25	Ischemic heart diseases			94,800	11.40%		<0.001	0.026
		2						101,848	12.30%			

**Table 8:** Measures of association for patients taking diltiazem and glioblastoma development

Cohort		Patients in cohort	Patients with outcome	Risk
1	Diltiazem	828,618	94	0.01%
2	No diltiazem	828,618	161	0.02%
		95% CI	z	p
Risk difference	-0.008	(-0.012, -0.004)	-4.196	<0.0001
Risk ratio	0.584	(0.453, 0.753)		
Odds ratio	0.584	(0.453, 0.753)		

Tables 9, 10 show the baseline characteristics and measures of association for our patients taking any of the aforementioned ion channel blockers. The combined cohort consisted of 1,576,042 patients; in this group, 184 patients (0.012%) developed glioblastoma. In a matched cohort of 1,576,042 patients not on any of these ion channel blockers, 287 patients (0.018%) developed a glioblastoma (p < 0.0001, OR = 0.641, 95% CI = 0.533, 0.771).

**Table 9:** Baseline characteristics for patients taking any of the ion channel blockers (verapamil, digoxin, amiodarone, or diltiazem)

Cohort 1 (N = 2,035,921) and cohort 2 (N = 6,089,750) characteristics before propensity score matching												
	Demographics											
		Cohort		Code		Mean ± SD		Patients	% of cohort		P-value	Std. diff.
		1		AI	Age at index	65.0 ± 15.9		1,998,460	100%		<0.001	1.14
		2				42.7 ± 22.6		6,089,598	100%			
		1		2106-3	White			1,415,666	70.80%		<0.001	0.083
		2						4,078,606	67.00%			
		1		2054-5	Black or African American			252,945	12.70%		<0.001	0.152
		2						1,102,736	18.10%			
		1		M	Male			1,023,976	51.20%		<0.001	0.21
		2						2,485,621	40.80%			
	Diagnosis											
		Cohort		Code		Mean ± SD		Patients	% of cohort		P-value	Std. diff.
		1		K74	Fibrosis and cirrhosis of the liver			27,381	1.40%		<0.001	0.048
		2						52,695	0.90%			
		1		I10-I16	Hypertensive diseases			849,956	42.50%		<0.001	0.396
		2						1,474,384	24.20%			
		1		E08-E13	Diabetes mellitus			361,253	18.10%		<0.001	0.195
		2						682,224	11.20%			
		1		N17-N19	Acute kidney failure and chronic kidney disease			298,367	14.90%		<0.001	0.346
		2						290,013	4.80%			
		1		F17	Nicotine dependence			171,097	8.60%		<0.001	0.044
		2						448,607	7.40%			
		1		F10.1	Alcohol abuse			37,767	1.90%		<0.001	0.045
		2						80,852	1.30%			
		1		I48	Atrial fibrillation and flutter			450,002	22.50%		<0.001	0.666
		2						113,082	1.90%			
		1		I20-I25	Ischemic heart diseases			486,062	24.30%		<0.001	0.546
		2						338,211	5.60%			
Cohort 1 (N = 1,576,042) and cohort 2 (N = 1,576,042) characteristics after propensity score matching												
	Demographics											
		Cohort		Code		Mean ± SD		Patients	% of cohort		P-value	Std. diff.
		1		AI	Age at index	62.3 ± 16.0		1,576,042	100%		<0.001	0.043
		2				63.0 ± 16.0		1,576,042	100%			
		1		2106-3	White			1,104,941	70.10%		0.041	0.002
		2						1,103,278	70.00%			
		1		2054-5	Black or African American			219,752	13.90%		<0.001	0.023
		2						232,607	14.80%			
		1		M	Male			773,458	49.10%		<0.001	0.01
		2						781,452	49.60%			
	Diagnosis											
		Cohort		Code		Mean ± SD		Patients	% of cohort		P-value	Std. diff.
		1		K74	Fibrosis and cirrhosis of the liver			19,910	1.30%		<0.001	0.013
		2						22,233	1.40%			
		1		I10-I16	Hypertensive diseases			582,928	37.00%		<0.001	0.063
		2						631,448	40.10%			
		1		E08-E13	Diabetes mellitus			253,554	16.10%		<0.001	0.033
		2						272,736	17.30%			
		1		N17-N19	Acute kidney failure and chronic kidney disease			173,596	11.00%		<0.001	0.037
		2						192,279	12.20%			
		1		F17	Nicotine dependence			130,924	8.30%		<0.001	0.013
		2						136,818	8.70%			
		1		F10.1	Alcohol abuse			27,412	1.70%		<0.001	0.013
		2						30,199	1.90%			
		1		I48	Atrial fibrillation and flutter			145,984	9.30%		<0.001	0.077
		2						112,626	7.10%			
		1		I20-I25	Ischemic heart diseases			279,011	17.70%		<0.001	0.05
		2						309,580	19.60%			

**Table 10:** Measures of association for patients taking any of the ion channel blockers (verapamil, digoxin, amiodarone, or diltiazem) and glioblastoma development

Cohort		Patients in cohort	Patients with outcome	Risk
1	Ion channel blockers (verapamil, digoxin, amiodarone, or diltiazem)	1,576,042	184	0.01%
2	No ion channel blockers (verapamil, digoxin, amiodarone, or diltiazem)	1,576,042	287	0.02%
		95% CI	z	p
Risk difference	-0.01%	(-0.009, -0.004)	-4.746	<0.0001
Risk ratio	0.641	(0.533, 0.771)		
Odds ratio	0.641	(0.533, 0.771)		

## Discussion

These results suggest that in patients using the ion channel blockers verapamil, amiodarone, or diltiazem, the odds of developing GBM were lower than in patients not taking these drugs. These results suggest a similar pattern for digoxin, albeit statistically insignificant. Furthermore, this association persisted when all patients were analyzed in a general group.

GBM originates from the brain's supporting cells, and these cells express a myriad of ion channels, including sodium, potassium, and anion channels [6]. Genomic analysis of mutations present in GBM has shown the presence of mutations in the genes encoding these ion channels in 90% of the glioblastoma samples examined [7]. Research suggests that mutations in these ion channels harbor a poor prognostic factor for patients by promoting proliferation, migration, and invasion of normal brain tissue by GBM. This effect is primarily believed to be mediated by the action of ion channels in promoting progression through the cell cycle [8].

Studies have shown that different types of Ca2+ selective ion channels are upregulated in GBM, where they confer a host of pro-survival benefits to the tumor, including promoting tumor invasiveness, proliferation, and resistance to apoptosis [9]. For example, diltiazem and verapamil primarily block the L-type voltage-gated calcium channels. This specific Ca2+ channel is expressed in several tumor cells, and blockage of this channel inhibits cancer cell invasion. This effect is primarily mediated by inhibiting the role of these channels in the development of filopodia, thereby preventing tumor cell migration and invasion of nearby healthy tissue [10]. Furthermore, verapamil has been shown to inhibit the T-type Ca2+ channels, and inhibition of this channel has been shown to induce apoptosis in glioblastoma cells [11]. As such, these Ca2+ channel blockers may prevent tumorigenesis via myriad mechanisms, including prevention of cell cycle progression, induction of apoptosis, and prevention of aberrant migration of malignant cells.

The anti-tumorigenic effects of cardiac glycosides have been previously established [12]. In addition, in vitro studies have shown that digoxin can exhibit antiproliferative and pro-apoptotic effects in GBM [13]. Although the mechanism of action has not yet been elucidated, the current school of thought suggests that inhibition of sodium currents might be a mechanism by which digoxin exerts its anti-tumor effects. Digoxin primarily acts by inhibiting the Na+/K+ ATPase, an energy-dependent transporter that plays a role in maintaining homeostatic levels of potassium and sodium in cells. Inhibition of this channel has been shown to independently induce cell death in GBM and increase tumor cells' sensitivity to chemotherapy [14]. As such, it is plausible that digoxin can play a role in preventing the development and progression of GBM.

K+ channels also contribute to the proliferation and apoptosis resistance exhibited by GBM. Specifically, GBM overexpresses certain voltage-dependent K+ channels, which are reportedly involved in signaling pathways that promote proliferation and inhibit apoptosis [15]. Some of these effects are caused by the role of K+ channels in establishing the resting membrane potential. Changes to this baseline can alter cell-cycle progression, promoting a pro-tumorigenic profile. Clinical studies have shown that the use of inhibitors of these channels is associated with better survival in patients with GBM, again emphasizing the role of these channels in the development and progression of GBM [16]. High expression of a specific subtype of the potassium channel (Kv10.1) in GBM cells is associated with a more dismal prognosis [17]. Amiodarone is an anti-arrhythmic that can block voltage-gated potassium, calcium, and sodium channels. This drug has also been shown to reduce glioblastoma growth in vivo by exhibiting direct anti-cancer effects and anti-angiogenic activity [18,19]. As such, some anti-tumorigenic effects of amiodarone are likely due to its inhibition of ion channels, which inhibit tumor cell proliferation and migration and its effect on angiogenesis.

Thus, the effect of these drugs on the development of GBM is probably due to a mixture of the various mechanisms aforementioned, including delayed progression across the cell cycle, inhibition of cell proliferation, and induction of apoptosis in de-novo malignant cells.

Several limitations exist in this study. Firstly, and most importantly, this analysis was primarily retrospective; hence, this investigation is limited to the constraints of such studies. Secondly, some information about the medication history could not be obtained from the TriNetX database. Specifically, the dosage of each medication, the indication in each patient, and the duration of usage of these medications could not be obtained. Furthermore, information about the stage and grade of each patient's GBM diagnosis could not be retrieved. The isocitrate dehydrogenase (IDH) mutation status and O6-methylguanine-DNA methyltransferase (MGMT) promoter methylation status of the tumors were unknown. The International Classification of Diseases, Tenth Revision (ICD-10) codes are primarily used for billing purposes. Finally, due to the nature of database studies, some misidentification is always present.

## Conclusions

These findings suggest that in patients taking the ion channel blockers diltiazem, amiodarone, or verapamil, the odds of developing GBM were lower than in patients not taking these drugs. The same relationship was seen in patients taking digoxin; however, this association was not statistically significant. Ion channels play a fundamental role in the development and progression of GBM. Therefore, inhibition of these channels could serve as a therapeutic target for the management of GBM.
